# Social Networks and Memory over 15 Years of Followup in a Cohort of Older Australians: Results from the Australian Longitudinal Study of Ageing

**DOI:** 10.1155/2012/856048

**Published:** 2012-08-29

**Authors:** Lynne C. Giles, Kaarin J. Anstey, Ruth B. Walker, Mary A. Luszcz

**Affiliations:** ^1^Discipline of Public Health, The University of Adelaide, Adelaide, SA 5005, Australia; ^2^Centre for Research on Ageing, Health and Wellbeing, The Australian National University, Building 63, Eggleston Road, Canberra, ACT 0200, Australia; ^3^SA Community Health Research Unit, Flinders University, G.P.O. Box 2100, Adelaide, SA 5001, Australia; ^4^Flinders Centre for Ageing Studies, Flinders University, G.P.O. Box 2100, Adelaide, SA 5001, Australia; ^5^School of Psychology, Flinders University, G.P.O. Box 2100, Adelaide, SA 5001, Australia

## Abstract

The purpose was to examine the relationship between different types of social networks and memory over 15 years of followup in a large cohort of older Australians who were cognitively intact at study baseline. Our specific aims were to investigate whether social networks were associated with memory, determine if different types of social networks had different relationships with memory, and examine if changes in memory over time differed according to types of social networks. We used five waves of data from the Australian Longitudinal Study of Ageing, and followed 706 participants with an average age of 78.6 years (SD 5.7) at baseline. The relationships between five types of social networks and changes in memory were assessed. The results suggested a gradient of effect; participants in the upper tertile of friends or overall social networks had better memory scores than those in the mid tertile, who in turn had better memory scores than participants in the lower tertile. There was evidence of a linear, but not quadratic, effect of time on memory, and an interaction between friends' social networks and time was apparent. Findings are discussed with respect to mechanisms that might explain the observed relationships between social networks and memory.

## 1. Introduction 

Over recent decades, there has been an accrual of evidence concerning the beneficial effects of social relationships on physical and mental health in older people, including longer survival [1], reduced risk of disability [2, 3], and reduced risk of dementia [4]. Cross-sectional [5] and longitudinal studies [6–11] have generally shown that older people with better social relationships also have higher levels of cognitive function. The influence of social relationships is broad.

In the conceptual model proposed by Berkman et al. [12], social *networks* underpin the ways in which social relationships affect health outcomes. Social networks were hypothesised by these authors to influence health through the provision of social support, social influence, social engagement and attachment, and access to material goods and resources. In turn, these aspects of social relationships affect health via behavioural and physiological pathways.

In the extant literature concerning cognitive function, there is considerable variability in the ways that social networks have been defined, which may partly explain differences in results across studies. For example, no association between overall social networks (defined as number of children, relatives, and friends seen at least monthly) and cognitive function was found in a recent cross-sectional study [5]. In a study of higher-functioning older persons [10], no effect of the total number of close social ties with children, relatives, and friend on cognitive performance at baseline and over 7.5 years of followup was observed. In contrast to these results, larger overall social networks have been demonstrated in other studies to be associated with a higher level of cognitive function at baseline [8] and with reduced rates of cognitive decline over 5 years [6] and 12 years of followup [8]. 

Different social roles are potentially fulfilled by different relationships with various people [13], and it has been argued that different types of social networks—that is, networks with children, relatives, friends, and confidants—may have differential effects on health [14]. For instance, there is longitudinal evidence of different impacts of network types on disability, residential relocation, and death [3, 15, 16]. Other authors have characterised the composition of individuals' social networks (diverse; friend, neighbour, or family focussed; restricted) and shown beneficial effects of diverse social networks on well-being [17, 18], physical activity [19], and survival [20]. However, relatively few studies have investigated the effects of different types of social networks on cognitive function. Zunzunegui et al. [11] showed less frequent contact with relatives, but not friends, was associated with cognitive decline over 4 years of followup. In a separate small study of 200 older adults, statistically significant effects of larger social networks with family and with friends were shown on global cognition over 5 years of followup [9]. However, participants with cognitive impairment at baseline were included in [11], so the possibility that poorer cognitive function leads to smaller social networks cannot be excluded as an explanation for the results. Furthermore, these results are equivocal with respect to the influence of different types of social networks, and followup duration was relatively short (4-5 years) in both studies. 

Based on the observation by Hughes et al. [9] that different cognitive domains show similar patterns of response to a variety of social resources, we selected episodic memory as the outcome measure in the present study (but see also [5]). The purpose of our study was to examine the relationship between different types of social networks and memory over 15 years in a large cohort of older Australians who were cognitively intact at study baseline. Our specific aims were to investigate whether social networks were associated with memory, determine if different types of social networks had differential effects on memory, and examine how any effects changed over time. 

## 2. Methods

### 2.1. Study Sample and Data Collection

We drew data from the Australian Longitudinal Study of Ageing (ALSA) that began in 1992 in Adelaide, South Australia. ALSA has been described in detail elsewhere [21]. ALSA's major objectives were to assess the effects of social, biomedical, behavioural, economic, and environmental factors upon age-related changes in the health and well-being of older persons [22]. The primary sample was randomly selected from the South Australian Electoral Roll, and stratified by local government area, gender, and age group (70–74, 75–79, 80–84, and ≥85 years). Older men were over-sampled to ensure sufficient numbers of males for longitudinal followup. Persons were eligible for the study if they were resident in the Adelaide Statistical Division and aged ≥70 years on December 31, 1992. 

We used five waves of data collection in the present study, taking all available data for the primary participants who completed an interview at baseline. Data relevant to the present study were collected at baseline, then at followup interviews approximately 2, 8, 11, and 15 years after baseline. The relevant ethics committee approved the study, and each participant (or their proxy) gave written informed consent at each wave. 

The Minimental State Examination (MMSE) was administered at each study wave and used as a dementia screen in the present study. Participants scoring below 24 at baseline were considered possibly cognitively impaired [23] and excluded from analyses to prevent inclusion of pre-clinical cases of dementia [24]. 

### 2.2. Episodic Memory

The outcome measure in the present study was an episodic memory measure calculated for each wave from a composite of recalled items that covered symbols, pictures, and words [25]. Recall of symbols from the Digit Symbol Substitution (DSS) subscale of the Wechsler Adult Intelligence Scale-Revised (WAIS-R; Wechsler 1991) was the basis for incidental symbol memory [26]. The total number of symbols correctly recalled out of a possible 9 was used as a measure of incidental symbol memory at each wave. A 15-item short form of the Boston Naming Task [27] gave a basis for the incidental picture recall measure in this study [26]. Participants were asked to recall the 15 pictures immediately after the task. Each participant was assigned a score based on the number of pictures they correctly recalled. The number of words correctly recalled from the three word-recall items of the MMSE was calculated for each participant, with a maximum possible score of three. The number of correctly recalled symbols, pictures, and words was then summed and each participant was assigned a memory score out of a maximum possible of 27. 

Picture and symbol components of the memory composite were completed as part of a separate clinical assessment conducted at each of the waves considered here, and not all participants agreed to take part in these further assessments. In total, at baseline there were memory composite scores from 706 participants who had an MMSE ≥ 24. 

### 2.3. Social Networks

Social networks with children, other relatives, friends, and confidants were hypothesised as predictors of memory. Measures of these four social network types were developed by Glass et al. [14] and have been previously validated for the ALSA sample using confirmatory factor analysis [28]. The children network combined information on the number of children, proximity of children, and frequency of personal and phone contact with children. The relatives network was calculated from the number of relatives (apart from spouse and children) the participant felt close to, and the frequency of personal and phone contact with these relatives. The friends network captured the number of close friends, personal contact, and phone contact. The confidant network reflected the existence of confidants and whether the confidant was a spouse. A total social network score was calculated as the sum of the children, relatives, friends, and confidant network scores. All component variables were standardized before the derivation of the social network variables. Each participant was then classified as being in the lower, mid, or upper tertile for each of the four social network types and the total social network variable according to the distribution of responses to each variable. These categorized network variables were used in subsequent analyses.

### 2.4. Demographic, Health, and Lifestyle Variables

To incorporate the effects of potential confounding variables in the analyses, a range of demographic and health variables were also considered. Age group (in five-year age bands), gender, current marital status (partnered or not), age left full-time education (≤ 14, > 14 years of age), number of chronic conditions (self-report, based on a list of 10 common conditions), mobility disability [29], depressive symptoms based on the CES-D scale [30] with cut-point of 17, alcohol use [31], and smoking status were considered in the adjusted analyses.

### 2.5. Statistical Analyses

We used all available data to fit random effects models [32] so as to assess the effects of each of the social network types on the memory composite over time. Such models characterize the overall pattern of change in the outcome, but allow for different coefficients for each individual, reflecting the correlation among observations for an individual [33]. 

We considered the effect of the different social network variables in separate models, and we also examined the interaction of the social network variables with time and time^2^ in the models. The most complex model included an interaction between linear (time) and quadratic (time^2^) functions of time in study and each type of social network, as well as the demographic, health, and lifestyle covariates indicated above. A similar approach was used by Ertel and colleagues [34]. A series of nested models that sequentially omitted the interaction between the time^2^ and social networks, time and social networks, and then the main effects of time^2^, time and social network variables were fit. We used likelihood ratio tests to compare between complex and simpler models. Stata version 12.1 was used in all analyses.

## 3. Results

As shown in Table 1, the average age at baseline of the 706 participants who were cognitively intact and had a baseline episodic memory score was close to 80 years; 18% of the cohort was initially aged ≥ 85 years. Two-thirds of the men in the study were married at baseline, while the majority of women were widowed. The prevalence of morbid conditions was relatively high, and the most common conditions were osteoarthritis, heart conditions, hypertension, diabetes, and cancers. More than one quarter of participants reported difficulty in climbing stairs or walking half a mile, and 12% of participants reported symptoms possibly indicative of clinical depression at the baseline interview. Five per cent of participants reported problem alcohol consumption, while more than half of the participants were current (7%) or ex-smokers (48%). The average times between baseline interview and each of the subsequent waves considered in the present study were 2.0 years (SD 0.1), 8.0 years (SD 0.2), 11.0 years (SD 0.2), and 15.2 years (SD 0.2), respectively.

**Figure 1:**
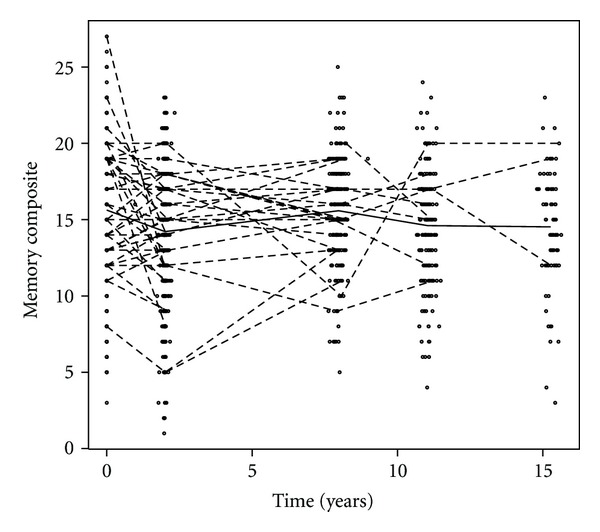
Trajectories of memory composite over fifteen years of followup in ALSA participants who were not cognitively impaired at baseline. Trajectories for a randomly selected 10% of participants are shown (connected dashed line segments). The mean memory composite score at each time of measurement is also shown (bold line).

Figure 1 presents trajectories of memory composite scores across 15 years of followup of ALSA participants. As is evident from this figure, there is considerable heterogeneity between participants in terms of memory over time. 

The random effects models showed that for the memory composite, there were significant main effects of time and total social networks. However, the interaction between time and total social networks was not statistically significant, suggesting that the changes in memory over time were parallel for the tertile groups of overall social networks. The coefficients in the models that adjusted for the range of demographic, health, and lifestyle covariates (shown in Table 2) demonstrate that the predicted memory composite scores of participants in the mid tertile of total social networks were 0.62 units (standard error (se) 0.22) higher than those in the lower tertile (Table 2). For those participants in the upper tertile of social networks versus the lower tertile, the effect was even larger (0.83, se 0.23). The effect of time was such that for every year, the memory composite scores declined by an estimated 0.15 (se 0.02) units. As shown in Table 2, these results were similar to those from the models which adjusted for sex and age group only. We also fit the final model for total social networks controlling for friends networks (see below), and the coefficients changed little from those presented in Table 2 (i.e., mid total social networks tertile *β* 0.66 se 0.27; upper total social networks tertile *β* 0.84; se 0.30 in the friends adjusted model). There was no evidence of a quadratic effect of time (either as a main effect or as an interaction with total social networks) on the memory composite variable. Separate analyses for males and females also showed broadly similar results for total social networks (Table 2), with effects of social networks on memory slightly larger for the women in our study than for the men. 

The suite of models that were fit investigating each type of social network showed that only the effect of friends networks on the memory composite was statistically significant. A significant interaction between friends networks and time was also observed. The interaction effect was such that the rate of decline in memory composite with every year (i.e., the slope) was steeper (0.25 units, se 0.04) for those in the lower tertile of friends networks, and the annual decline in memory composite was less for those in the mid tertile (−0.14 (i.e., −0.25 + 0.11), se 0.05) and upper tertile (−0.15, se 0.05) of friends networks. Separate analyses for males and females suggested the effect of friends social networks differed between the sexes, with a larger effect of friends social networks observed for females than for males. There were no statistically significant effects of the children, relatives, or confidants social networks at baseline on memory in our study, either as main effects or in interaction with time or time^2^.

## 4. Discussion

We have demonstrated that larger friends social networks and overall social networks had significant benefits for memory in a population-based cohort of participants who were cognitively intact at baseline and followed for an average of 15 years. The results suggested a gradient in the effect of social networks, so that participants in the upper tertile of friends or total social networks had better memory scores than those in the mid tertile, who in turn had better memory scores than participants in the lower tertile of social network. The results also suggested that the observed effects of total and friends social networks were slightly larger for females than for males. Notably, we did not find any significant effects of social networks with children, relatives, or confidants on memory. The five occasions of measurement also allowed us to estimate the rate of decline, which showed that while consistent, the effect of time is small. Our findings point to the importance of disaggregating kin and nonkin networks, rather than considering only aggregate measures of social networks that do not distinguish between different types of social ties.

The mechanisms through which different types of social networks affect cognitive function remain unclear. Berkman et al. [12] contend that social networks influence health in four main ways, through the provision of social support, social influence, social engagement and attachment, and access to material goods and resources. In turn, these psychosocial mechanisms influence health through health behavioural, psychological, and physiological pathways. For memory specifically, social networks and memory may be related in several ways. Social networks are the basis for social engagement, which is cognitively stimulating and may enhance neural plasticity in ageing, thereby maintaining cognitive reserve [35]. Thus better social networks might lead to continued psychological stimulation, delaying cognitive decline, or impairment. An alternative possible mechanism is that the stronger social networks may serve to buffer against stress, through modifying its effects on the activation of the hypothalamic-pituitary-adrenal axis of the central nervous system [12]. This affects neuronal functioning, and in this way individuals with better social networks are protected from some of these neuroendocrine processes [36]. Another possibility is that social networks facilitate access to health care, indirectly forestalling brain pathology and other disease processes that affect cognition [37, 38]. Finally, it is also possible that changes in cognitive function affect social networks. Cognitive impairment may lead to withdrawal from social activities, because of difficulties in participation or in maintaining relationships. These mechanisms could apply equally to friends and total social networks. Reasons for why other types of social networks are not as beneficial remain unclear.

The finding in the present study that social networks with friends had specific effects on memory suggests other ways that social relationships may promote cognitive function. Friends may encourage health seeking and health promoting behaviour, such as physical activity, which may in turn have beneficial sequelae for cognitive health. It is possible that health advice is better received by individuals when it is offered by friends, rather than family or confidants. It is well established that friends can have effects on other psychological measures including depression, self-efficacy, self-esteem [39], coping and morale [18], and sense of personal control [40]. It is possible that these effects are due to the reinforcement of social roles, or because interactions with friends can become increasingly discretionary with age [41]. The friendship networks that are retained in late life may offer high levels of socioemotional support, and thus confer benefit to individuals. It is also possible that less discretionary social networks, such as those with children and relatives, do not only involve positive social interactions, and interactions involving conflict may negate any beneficial effects on cognitive function. However, there is some evidence that negative social interactions are associated with better cognitive ability [9, 10], possibly due to greater cognitive stimulation from negative interactions. Further research to disentangle the mechanisms through which social networks with kin and nonkin affect health in later life is clearly warranted. Further work with the ALSA databank is also needed to investigate if the observed relationships between memory and social networks hold across other measures of cognitive function. 

The findings from this study must be interpreted with some caveats borne in mind. While a range of potential confounders were included in the analyses, we cannot discount the possibility that residual confounding could have affected our results, as not all aspects of social engagement or lifestyle were taken into account. Furthermore, ALSA was not explicitly designed to examine the effects of social networks on memory, and the analyses are based on self-reported predictor variables and covariates measured at baseline. We also cannot discount the possibility that for some participants, previous declines in memory and other aspects of cognitive function led to lower social networks scores at baseline. In turn, these lower baseline social networks may then be associated with lower memory scores at subsequent followup times. Social networks may change over time, but the social networks considered in the present study were defined using only baseline data and so cannot reflect social networks at other points—either earlier or later—in the life course. However, total network size has been demonstrated as relatively stable over a long followup period in a study of older Dutch people [42]. Alternative derivations of the memory composite measure (e.g., with differential weightings of the items in the measure or with data reduction techniques such as principal components analysis) could have been applied. However, the interpretation of results based on more complex composite measures is more difficult, off-setting any potential advantages from such derivations of a memory measure. The nonrespondents to ALSA may also have been more socially isolated than participants, although nonresponse bias has generally been demonstrated as minimal in other analyses of ALSA data [26, 43]. We did not explicitly model the risk of dropout or death in this study. A recent study concerning education and cognitive decline [44] suggests this modelling approach may be a worthy avenue for future investigation in this area. A final point is that the MMSE is a crude screening tool for dementia. However, it is very widely used and while it may not eliminate all those with preclinical dementia, the inclusion of participants with possible cognitive impairment in the study would only have added to the variation in observed memory scores. This would serve to attenuate any true association between social networks and memory, so it is likely that the associations we observed would also be found in a more strictly cognitively intact sample. It must be noted, however, that the limitations identified above are true of the majority of longitudinal studies that have considered social relationships and cognitive function in older adults. 

We believe these restrictions are balanced by ALSA's strengths, which include the richness of the data, the Australian setting, and the inclusion of residents in aged care facilities. ALSA also included a more heterogeneous population-based sample than many other longitudinal studies of ageing. An additional strength is that the present findings are drawn from five repeated measurements of memory, spanning 15 years of followup, which allowed us to examine whether there was evidence of a nonlinear relationship between cognitive function and time. We have not identified any other studies that considered social networks and memory with as many repeated assessments or the duration of followup that we have presented.

In summary, we have shown that friends and total social networks are associated with memory over 15 years of followup in a large cohort of older Australian men and women who were cognitively intact at baseline. Having a larger social network with more frequent contacts, especially with friends, appears important for preserving cognitive function and slowing the rate of decline.

## Figures and Tables

**Table 1:** Summary statistics for 706 participants with no cognitive impairment at baseline of ALSA.

Characteristic	Summary^1^
Age years mean (SD^2^)	78.6 (5.7)
Gender	
Male	476 (67.9)
Female	230 (32.1)
Place of residence	
Community	673 (95.3)
Institution	33 (4.7)
Marital status	
Married/De facto	413 (58.5)
Widowed	244 (34.6)
Single	49 (6.9)
Age left school	
≤14 years	360 (51.0)
>14 years	346 (49.0)
Number morbid conditions mean (SD)	1.6 (1.1)
Mobility	
No disability	512 (72.3)
Disability	194 (27.7)
Depressive symptoms	
CES-D < 17	621 (88.0)
CES-D ≥ 17	85 (12.0)
Alcohol problem	
AUDIT score < 8	671 (95.0)
AUDIT score ≥ 8	35 (5.0)
Smoking status	
Never smoker	320 (45.3)
Ex smoker	340 (48.2)
Current smoker	46 (6.5)
Children social network score mean (SD)	0.05 (0.75)
Relatives social network score mean (SD)	0.02 (0.77)
Friends social network score mean (SD)	0.13 (0.74)
Confidants social network score mean (SD)	0.08 (0.75)
Total social network score mean (SD)	0.27 (1.64)
Mini-mental state exam mean (SD)	28.3 (1.7)
Memory composite mean (SD)	15.7 (3.4)

^1^Shown is the number (%) of participants unless otherwise indicated.

^2^SD is standard deviation.

**Table 2:** Summary of effects of friends and total social networks on cognitive function^1^.

Covariate						Males			Females			
	*β* ^ 1^	se^2^	*P*-value	*β* ^ 3^	se	*P*-value	*β* ^ 3^	se	*P*-value	*β* ^ 3^	se	*P*-value
Total^1^												
Time	−0.16	0.02	<0.001	−0.15	0.02	<0.001	−0.17	0.02	<0.001	−0.15	0.04	<0.001
Network mid tertile	0.77	0.26	0.004	0.62	0.22	0.005	0.59	0.30	0.052	1.00	0.49	0.040
Network upper tertile	1.09	0.26	<0.001	0.83	0.23	<0.001	0.68	0.31	0.029	1.38	0.71	0.004
Friends^1^												
Time	−0.23	0.04	<0.001	−0.25	0.04	<0.001	−0.21	0.04	<0.001	−0.38	0.10	0.001
Friends mid tertile	0.13	0.28	0.646	0.00	0.28	0.986	0.51	0.32	0.112	−0.99	0.55	0.073
Friends upper tertile	0.38	0.28	0.170	0.25	0.28	0.359	0.37	0.32	0.246	−0.03	0.54	0.959
Friends mid tertile × time	0.10	0.05	0.052	0.11	0.05	0.034	0.05	0.06	0.418	0.32	0.12	0.010
Friends upper tertile × time	0.08	0.05	0.107	0.10	0.05	0.054	0.07	0.06	0.193	0.22	0.11	0.051

^1^Model also includes sex, age group.

^2^se is standard error.

^3^Model also includes sex, age group, education, marital status, disability status, chronic conditions, depressive symptoms, alcohol consumption and smoking status.
